# Neural correlates of free recall of “famous events” in a “hypermnestic” individual as compared to an age- and education-matched reference group

**DOI:** 10.1186/s12868-018-0435-y

**Published:** 2018-06-19

**Authors:** Thorsten Fehr, Angelica Staniloiu, Hans J. Markowitsch, Peter Erhard, Manfred Herrmann

**Affiliations:** 1 Center for Cognitive Sciences, University of Bremen, Bremen, Germany; 2 University of Bremen, Hochschulring 18, 28359 Bremen, Germany; 3 Center for Advanced Imaging, Universities of Bremen and Magdeburg, Bremen, Germany; 4 Physiological Psychology, University of Bielefeld, Bielefeld, Germany; 5 AG in vivo MR, University of Bremen, Bremen, Germany; 6 Hanse Institute for Advanced Study (HWK), Delmenhorst, Germany

**Keywords:** Memory, fMRI, Superior memory, Memory strategy, Experts, Complex cognition

## Abstract

**Background:**

Memory performance of an individual (within the age range: 50–55 years old) showing superior memory abilities (protagonist PR) was compared to an age- and education-matched reference group in a historical facts (“famous events”) retrieval task.

**Results:**

Contrasting task versus baseline performance both PR and the reference group showed fMRI activation patterns in parietal and occipital brain regions. The reference group additionally demonstrated activation patterns in cingulate gyrus, whereas PR showed additional widespread activation patterns comprising frontal and cerebellar brain regions. The direct comparison between PR and the reference group revealed larger fMRI contrasts for PR in right frontal, superior temporal and cerebellar brain regions.

**Conclusions:**

It was concluded that PR generally recruits brain regions as normal memory performers do, but in a more elaborate way, and furthermore, that he applied a memory-strategy that potentially includes executively driven multi-modal transcoding of information and recruitment of implicit memory resources.

**Electronic supplementary material:**

The online version of this article (10.1186/s12868-018-0435-y) contains supplementary material, which is available to authorized users.

## Background

Aside from the time-based distinction into short-term/working and long-term memory [1], memory is nowadays partitioned into content-based systems [2, 3]. Long term memory (LTM) has been discussed to be distinguished into episodic-autobiographical memory (memory for personal events or experiences), semantic memory (conscious knowledge of facts, including factual self-knowledge), perceptual memory (conscious familiarity judgments), procedural memory (mechanical, motor-related skills) and priming (higher likelihood of re-identifying previously perceived stimuli) [4–6]. Superior LTM performance has been described to tap into different memory systems. There are reports of individuals with highly superior autobiographical memory [7, 8], of semantic memory experts [9–11], and descriptions of specific types of hypermnesia [12, 13].

In general, LTM performance might be facilitated by the application of optimal learning strategies [14, 15] and/or the existence of elaborated, domain-specific expert knowledge networks [16], to which new information can be both efficiently associated to and recalled from. Parker et al. [7] proposed a specific form of superior memory performance, the hyperthymestic syndrome (HS), which is characterized by superior memory that is assumed to be automatically organized, and not based on explicit mnemonic strategies. It addresses idiosyncratic memory domains; these particular individuals do not necessarily score higher than average on standard memory tests tapping on information that is irrelevant for them. In a similar way, Norman Brown [17] put forth a model of “historical memory” elaboration that assumes an association of historical facts of public interest to individually relevant episodic information, which might be related to both the template theory proposed by Gobet and Clarkson [16] and, in case of superior performance, to the HS advanced by Parker et al. [7].

The neural mechanisms for memory encoding and retrieval are still under debate, and especially for superior memory performance, they are still largely unknown. In particular, since the prominent patient H.M. showed selective impairment in consciously encoding and consolidating new facts and events long-term [18–20], these processes were assumed to be mediated by the hippocampus and its adjacent medial temporal brain regions [21–23]. However, other regions of the limbic system situated in medial diencephalon and basal forebrain, equally contribute to these processes [5]. There are also theories suggesting that LTM networks can be associated with highly integrated networks distributed all over the neural system [24–26] and that the hippocampal formation is involved in both conscious and unconscious information processing [27, 28].

Functional neuroimaging and clinical studies involving patients with different forms of amnesia as a result of regional brain damage, however, support the idea that different memory systems might recruit at least partially “distinct” brain networks [5, 20, 29]. Aside from limbic structures involved in processes of binding and associating information to LTM, further cortical structures—sometimes referred to as expanded or greater limbic system [30, 31]—such as the retrosplenial cortex and the precuneus were related to processes of imagination, of the representation of memories, and to familiarity [32–34]. These structures might play an important role in both normal, but in particular, in superior LTM performance.

In the present study, we carried out a comprehensive neuropsychological assessment and additionally conducted a functional magnetic resonance imaging (fMRI) study that focussed on a memory retrieval task in an individual (PR) with superior historical facts knowledge (“famous events memory”). The present experimental design was specifically designed to examine the neural correlates of recalling memories related to historical facts. FMRI-contrast included the conditions ‘recall of declarative memory content WITH reference to historical facts’ and ‘recall of declarative memory content WITHOUT reference to historical facts’. It was assumed that PR should show less pronounced activation patterns in frontal (i.e., executive memory organization), but enhanced neural involvement of limbic brain regions (automatic and/or pre-attentive memory organisation) and precuneus (perceptual, imaginative, confidence judgments and/or familiarity based memory strategies) in comparison to an age- and education-matched reference group.

## Results

### Behavioural data

**Fig. 1:**
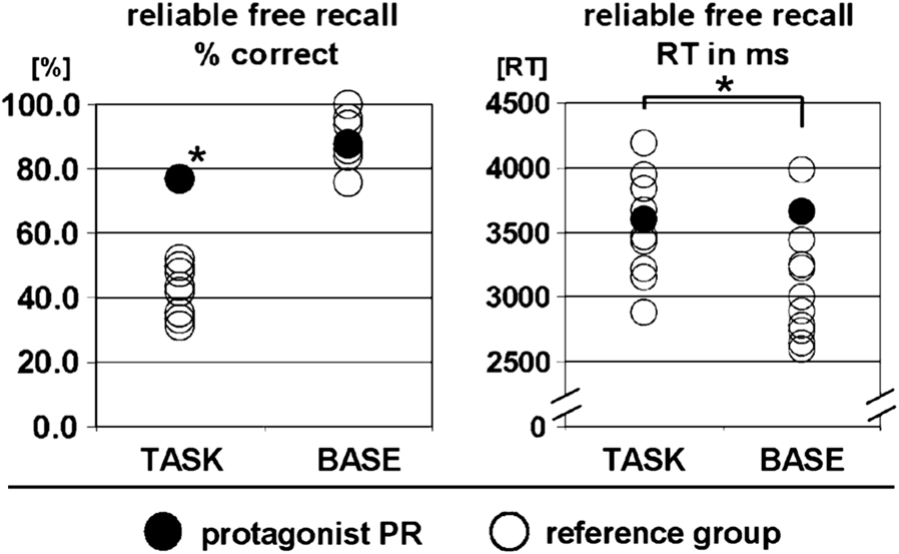
Memory performance in percentage of correct freely recalled answers (left panel) and the respective mean response times (right panel); asterisks indicate significant differences (*p* < .05; details, see text) and whiskers indicate standard deviations

Before applying parametric t-statistics, a Shapiro–Wilk-test (S–W-T) was performed in order to test, whether variables of interest were normal-distributed. In the reference group, the percentage of freely recalled correct answers (consistently shown for both scanner session and post hoc debriefing) was significantly higher in the BASE (test on normal distribution: S–W-T, W = .93, *p* = .45) compared to the TASK (test on normal distribution: S–W-T, W = .88, *p* = .13)-condition (TASK: 41.7 ± 8.3%; BASE: 89.6 ± 7.2%; *t* Test: t = 15.2, *p* < .001; see Fig. 1a), and reference group members performed better than chance level in both TASK- and BASE-conditions (TASK vs. 25%: t = − 1.9, *p* < .05; BASE vs. 25%: t = − 8.6, *p* < .001 [35, 36]). Compared to the reference group, PR showed a higher correct percentage rate in the TASK-condition (t = 4.1, *p* < .01 [35, 36]; see Fig. 1a) and comparable performance in the BASE-condition. All participants showed high performance in the BASE task. As, however, performance-values were normal-distributed (see above) and PR's performance ranged in the middle of the reference-group performances, we rather tend to disclose a ceiling effect. Participants in the reference group showed longer response times for correctly answered TASK-trials (test on normal distribution: S–W-T, W = .87, *p* = .09) compared to BASE-trials (test on normal distribution: S–W-T, W = .90, *p* = .24) during free recall (TASK: 3728 ± 733 ms; BASE: 3056 ± 429 ms; t = 4.4, *p* < .01; see Fig. 1b). According to Crawford and Howell [35], PR (TASK: 3599 ms; BASE: 3664 ms) did not differ in any response time value from the reference group.

### FMRI-data: TASK versus BASE-condition in PR and in reference participants

**Fig. 2:**
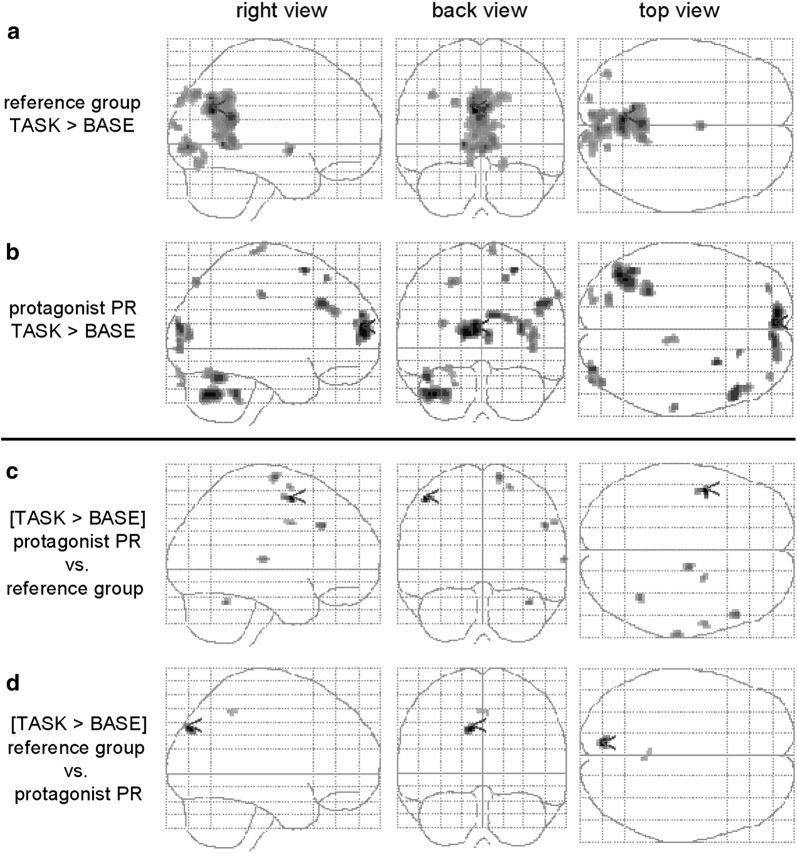
Glass-brain views of the contrasts TASK versus BASE for (**a**) the reference group, (**b**) the protagonist PR, (**c**) protagonist PR versus reference group and (**d**) vice versa (details for statistical procedures, see methods section). The respective anatomical regions, MNI to Talairach transformed coordinates and t-values were listed in Table 1 (columns A–D). All contrasts: *p* < .001

For the reference group, second level fMRI-analysis for TASK in contrast to BASE-condition revealed activation clusters in the right anterior and left posterior cingulate, bilateral precuneus, cuneus, and lingual gyrus (Fig. 2a; Table 1, column A). Protagonist PR showed activation clusters in widespread bilateral superior, medial, and middle frontal gyri, right postcentral gyrus, left precuneus, right middle and left inferior occipital gyri, left fusiform gyrus, and left cerebellar regions for the contrast TASK versus BASE-condition (Fig. 2b; Table 1, column B).

**Table 1:** Anatomical regions, peak activation t-values, and Talairach-coordinates for contrasts between TASK- and BASE-conditions (arranged in columns A–D) separately for (column A) the reference group (RG, one-sample t-test), (column B) the protagonist (PR, first level contrast), (column C) PR versus RG, and (column D) RG versus PR (latter two comparisons according to Crawford and Garthwaite [42, 43]

Anatomical region	H	A				B				C				D			
		Ref. group				Protagonist				TASK > BASE				TASK > BASE			
		TASK > BASE				TASK > BASE				PR > RG				RG > PR			
		t	x	y	z	t	x	y	z	t	x	y	z	t	x	y	z
*Peak activations: TASK versus BASE conditions*																	
Precentral Gyrus	R									5.7	57	2	33				
Superior Frontal Gyrus	L L R R R R					4.3 3.6 4.2 3.8 3.7 3.4	− 12 − 32 10 22 20 38	59 51 57 59 30 53	8 16 21 17 48 16	7.4	14	− 8	67				
Medial Frontal Gyrus	L R R					4.4 3.9 3.5	− 4 6 8	57 55 − 22	12 6 70								
Middle Frontal Gyrus	L R R R					4.2 3.7 4.0	50 46 26	27 36 14	30 24 55	9.9 7.1 5.9	− 46 50 22	5 27 3	51 30 59				
Paracentral Lobule	L													4.7	− 4	− 40	48
Anterior Cingulate	R	8.2	2	− 2	− 5												
Posterior Cingulate	L	10.3	− 2	− 47	23												
Postcentral Gyrus	R					3.6	61	− 21	40								
Precuneus	L L L R R	12.1 6.3 5.3 7.0 4.6	− 6 − 40 − 6 4 2	− 61 − 74 − 80 − 72 − 81	29 41 41 40 41	3.6	− 24	− 71	51					5.1	4	− 46	47
Middle Occipital Gyrus	R R R					4.0 3.7 3.7	32 42 42	− 85 − 79 − 87	19 13 6								
Inferior Occipital Gyrus	L					3.3	− 24	− 88	− 12								
Cuneus	L R R	6.3 6.2 6.1	− 20 10 16	− 88 − 90 − 84	36 17 37									11.0	− 8	− 78	37
Lingual Gyrus	L L L R R	9.6 7.2 6.9 8.3 6.0	− 14 − 8 − 6 4 18	− 54 − 85 − 78 − 81 − 76	3 1 1 2 − 8												
Fusiform Gyrus	L					4.0	− 44	− 57	− 17								
Superior Temporal Gyrus	R									6.1	65	− 21	8				
Cerebellum/Tuber	L					4.2	− 38	− 62	− 27								
Cerebellum/Culmen	L					3.9	− 30	− 44	− 28	6.3	38	− 51	− 19				
Cerebellum/Declive	L					3.4	− 22	− 86	− 19								

H = hemisphere: L = left, R = right, all statistics *p* < .001, uncorrected, minimum voxel cluster size k = 10 voxels

All PSC-value-distributions were tested for deviations from normal distribution via Shapiro–Wilk-Test: rPCG: W = .92, *p* = .33; rSFG: W = .86, *p* = .05; lMFG: W = .98, *p* = .96; first cluster rMFG: W = .94, *p* = .48; second cluster rMFG: .93, *p* = .42; rSTG: W = .94, *p* = .47; rCUL: W = .92, *p* = .29. The direct comparison between PR and reference group yielded larger contrasts between TASK- and BASE-conditions distributed over precentral gyrus, right superior and bilateral middle frontal gyri, right superior temporal gyrus, and the left culmen (Fig. 2c; Table 1, column C). Reference group versus PR showed larger contrasts in left paracentral lobule, right precuneus, and left cuneus (Fig. 2d; Table 1, column D).

**Fig. 3:**
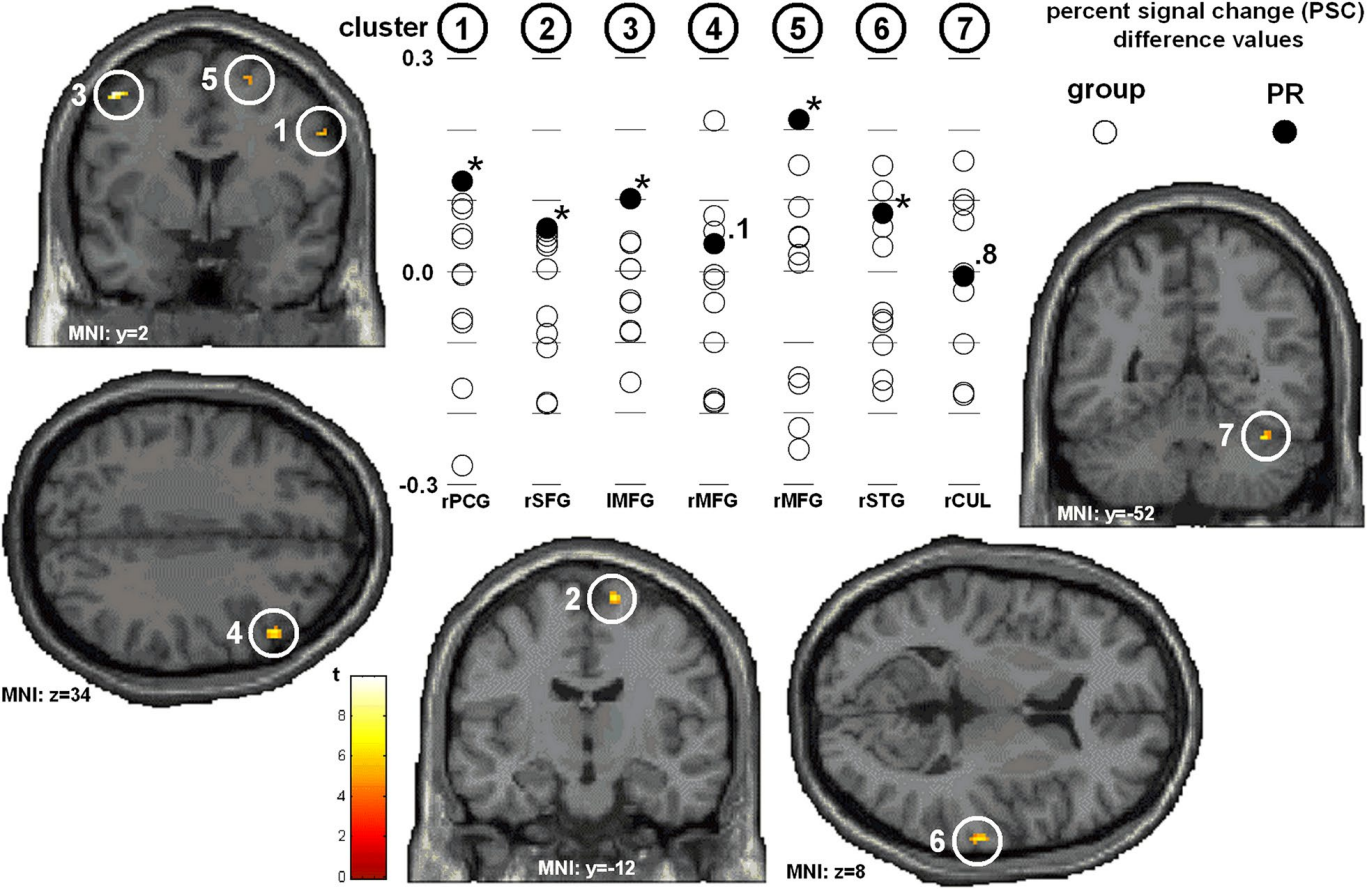
Regions of interest (ROI-) analyses showing section views of seven ROIs. Box-plots illustrate the distribution of percent signal change (PSC) difference values (TASK—BASE) for the reference group and black dots indicate the respective values for protagonist PR; rPCG = right PreCentral Gyrus, rSFG = right Superior Frontal Gyrus; lMFG = left Middle Frontal Gyrus; rMFG = right Middle Frontal Gyrus; rSTG = right Superior Temporal Gyrus; rCUL = right CULmen

In Fig. 3, distributions of difference-values between PSC-values for TASK and BASE-conditions for several ROIs were illustrated for the reference group and separately for PR. T-Test for single means yielded to individual versus group differences in right precentral gyrus, right superior frontal gyrus, left and right middle frontal gyri, and right superior temporal gyrus.

## Discussion

In the present study, functional neuroimaging was used to compare a protagonist’s (PR) superior memory performance in a famous events free retrieval task (contrasted with a semantic non-historical facts free retrieval task serving as baseline) with the performance of a reference group. PR performed significantly better than the reference group and showed activation patterns predominantly distributed over frontal and cerebellar, but also in parietal, occipital and occipito-temporal brain regions. The reference group demonstrated activation patterns in the cingulate cortex, parietal, and occipital brain regions. The direct comparison between PR and the reference group confirmed larger contrasts for frontal and cerebellar regions in PR and for parietal and occipital brain areas in the reference group. It appears that PR predominantly recruited right hemispheric frontal resources potentially related to his superior memory retrieval performance [44, 45].

### Which type of expert is PR?

First, PR showed superior memory performance for famous events retrieval, and he scored predominantly at average or above average in most retrograde and anterograde memory tests in the neuropsychological assessment. Only for complex anterograde non-verbal memory processing he scored below average. Incidentally, the case described by Parker et al. [7] also showed impairments in anterograde non-verbal memory tasks. Thus, while PR shares certain similitudes with HS (hyperthymestic syndrome [7]), he presents with unique features, which deviate from the HS prototype. He showed, beside superior historical facts memory, deviations from standards in non-idiosyncratically eminent memory domains. The mnemonic performance of PR can be at least partly understood within the model of Markowitsch and Tulving. Incidentally, Tulving [46] indirectly anticipated later models of mnemonic processing, which describe porous boundaries between memory systems [47]. In 1995, Tulving [46] proposed his SPI-model which states that encoding of information follows a regular sequence—that means it is serial in that way that first simple, implicitly functioning, memory systems are engaged and only at the end of the series explicit, episodic encoding occurs. Information then is stored in parallel memory systems in the brain and it can be retrieved independently from the systems used for the encoding process (SPI = Serial, Parallel, Independent). As PR describes his knowledge as “popping out automatically”, it can be speculated that according to Tulving’s [46] SPI-model, PR retrieves his knowledge “automatically”, and therefore more independently from his initial encoding of it, than it likely is the case in most human beings.

In addition, PR showed sub-average performance on tests for executive functions. While this type of performance may be interpreted as a hint towards a savant-syndrome [48], there was no clear clinical, neuropsychological or anamnestic evidence in his case that he fell into this category. He showed a superior intelligence level comparable to the reference group as confirmed by a verbal intelligence test. Furthermore, he scored above average on standardized laboratory tests for assessing social cognition and emotional processing, although he reported some interpersonal difficulties in real-life, which were being addressed in psychotherapy. Incidentally, the case described by Parker et al. [7] also presented with some difficulties in the executive functioning domain. Furthermore, LePort et al. [8] found in a case series of patients with highly superior autobiographical memory abilities a psychological profile indicative of obsessive compulsive tendencies. These tendencies typically are accompanied by a reduction in cognitive flexibility, which gets translated in impaired performance on corresponding neuropsychological tasks. The deficient performance of PR on tasks tapping on cognitive flexibility and the anamnestic reports about PR may speak in favour of obsessive compulsive personality phenotypic traits. These traits may promote an automatic engagement in repetition of mnemonic material of special interest, with consequences for processes of encoding and consolidation. Conclusively, PR cannot be seen comparable to individuals with HS or to savants in the classical sense. It rather appears that he is an expert sharing some features of HS and savant people, but does not fully overlap with any of these prototypes, displaying unique features. In the following, characteristics in fMRI activation patterns are discussed to conclude about the individual mnemonic strategy that PR might have applied to score higher in free historical facts retrieval than the reference group.

### Can the present functional neuroimaging data explain PR´s profile of superior memory performance?

The hypothesis that PR should show rather posterior and/or subcortical instead of frontal activation patterns when compared to the reference group, had to be rejected. The opposite was the case: PR showed predominantly right frontal patterns of larger contrasts between free historical and non-historical semantic facts retrieval. At first glance, this finding appears quite disillusioning in view of the hypotheses postulated in the introduction section, however, combined with the data that PR also showed remarkable cerebellar recruitment, a hybrid mental strategy of memory processing including both explicit and implicit components might be conceivable [10, 48]. And, this argument also goes with the hybrid classification (between savant and expert) concluded from the neuropsychological testing data as mentioned in the previous section.

### Level of processing and multi-modal integration of memory

There were also exclusive activation patterns in PR not present in the reference group and vice versa, which could in part only be interpreted in an individual (PR) or group-internal (reference-group) way as the direct comparison between PR and the reference group did not reach statistical significance in the respective brain regions. In posterior brain regions, associated with perceptual information processing, PR showed activation patterns rather adjacent to primary (i.e., middle and inferior occipital gyrus, and postcentral gyrus) and less in hetero-modal, medial (i.e., cuneus and lingual gyrus) cortical brain regions as the reference group did. Rather medial activation patterns in the reference group might be related to a higher level complex or abstract memory processing [25]. This finding might also explain why PR scored above average in simple, but below average in complex recognition tasks that usually require a deeper associative memory strategy.

Transcoding and integration of information across processing modalities (i.e., implicit and explicit processing, and verbal, visual, and spatial modalities, etc.), as can be seen for example in synesthesia [49–51] was discussed as a potential feature of both superior expert and savant memory performance involving occipito-temporal regions such as the fusiform gyrus [9, 10]. The present data showed exclusive fusiform gyrus involvement in PR, but not in the reference group. It should however be mentioned that the direct comparison between groups via between-group t-test did not reveal differences in this region. Nevertheless, in combination with an exclusive cerebellar recruitment in PR, this data might point to an implicit-explicit (and vice versa) memory transcoding strategy facilitating superior memory performance in historical facts retrieval, but also in other simple memory processing domains as shown by the neuropsychological assessment.

The stronger involvement of superior temporal gyrus (STG) in PR additionally supports the idea of pronounced multi-modal integration of information in LTM potentially facilitating his retrieval performance. The multi-associative nature of STG has been documented by studies discussing the role of the STG in retrieving both autobiographical events [52–54] and semantic facts, such as public events or “long-established knowledge about the world” [52, 55, 56].

Furthermore, the right superior temporal cortex was thought to be engaged in spatial awareness and exploration [57]. Along this line of argumentation, the stronger recruitment of STG in PR may signify a strategy to navigate through personal past by making use of more elaborated spatial exploration strategies [53]. Recently, Manning and colleagues argued that public semantic memory is supported by both the semantic and episodic memory system [58]. The stronger involvement of the STG in PR in contrast to the reference group might again point to a particular engagement of the episodic memory system in public semantic knowledge processing in the case of PR and other individuals with highly superior autobiographical talents [8, 59–61].

### Limitations of the present study

In future studies the number of tasks in the different task-condition should be perfectly matched.

For the present reference group there were 15–25 correct response trials in the TASK-condition left to be modeled for the respective fMRI-statistics. Despite normal distribution and despite numerous studies also reporting reliable data based on a quite low number of trials and/or individuals, this point should be carefully considered for an appropriate interpretation of the here presented data. In future studies additional measurement sessions should be taken into account to extend the number of valid trials. In the present study, we were however forced to balance the available time (PR was only available for a short time period) and test-statistical requirements.

## Conclusions

Specific complex mental processes cannot be inferred directly from functional brain imaging data [62], however, there is evidence that regional brain activation can help to understand the underlying mental principles involved in a certain complex mental process such as the applied visual, verbal or spatial modality, or perceptual and/or executive processing types, and others more [63–65]. It appears that the utilization of individual mental strategies also plays an important role in effective memory processing [61, 66–69]. And, these individual memory strategies can be modulated by executive mental processing as potentially reflected in the pronounced recruitment of frontal brain regions in PR. Furthermore, the present data support the idea that superior mental processing in experts can be facilitated by the conceptually driven recruitment of implicit/procedural memory resources (i.e., potentially reflected by the involvement of cerebellar brain areas [10]). A more detailed assessment of mental strategies in individuals with superior mental performance can provide insights into effective implicit memory usage potentially driven by explicit executive mental processing. Functional neuroimaging can help to evaluate the recruitment of implicit mental resources that are difficult to be assessed by explicit surveys.

## Methods

### Study participants

The individual protagonist PR (within the age range: 50–55 years old) was a healthy, right handed expert with superior memory abilities.

Applying a test-battery several months before the fMRI-measurement session provided a detailed neuropsychological performance-profile of PR (see Table 2 for details).

**Table 2:** Protagonist PR was examined with a battery of different test inventories

Mental domains and tests	Score	Interpretation
*Attention, concentration*		
Trail making test A + B	A: 42 s, 1 error; B: 174 s, 0 errors	Below average
d2-R test	122 correct, 12 errors	Average
WMS-R, attention and concentration index	96	Average
*Intelligence*		
Mehrfach–Wahl–Wortschatz-test B	34 of 37 (IQ 130)	Above average
Wechsler intelligence test raw scores	24, 23, 15, 34 (IQ > 125)	
*Visuo-constructive abilities*		
Rey–Osterrieth figure (ROF), copy	36	Normal
*Interference*		
Color-word-interference test (CWIT)	12, 22, 37 s	Above average
*Anterograde memory*		
ROF, by heart after ½ h	21	Average
WMS-R, general memory	94	Average
Verbal learning memory test (VLMT)	63 learning, 7 interference; 15 + 15 in Trials 6 + 7, 50/0 in recognition	Above average
Doors test	simple recognition: A = 12; complex recognition: B = 5	Above average Below average
*Retrograde memory*		
Semantic: Famous Faces Test (38 pictures)	30 directly identified, 2 with cues	Above average
Episodic-autobiographical old memory (EAMT)	Gives per epoch well-described examples	Very good
Semantic old famous events (1970s–1990s)	22 named, 1 recognized, 2 unknown	Very good
*Emotion*		
Mind in the Eyes Test	19/24 correct	Above average
Florida Affect Battery	Facial Identity Discrimination: 20 of 20 Facial Affect Discrimination: 20 of 20	Above average Above average
*Problem solving ability, cognitive flexibility, executive functions, risk taking behavior*		
Cronin–Golomb concept formation task	15–16 of 17	Good average
Category test	4, 5, 5 categories	Below average
Tower of Hanoi (4 discs)	49 moves, 5 min, 21 s	Below average
Word fluency (COWAT Test)	17 + 10 + 14	Average
Wisconsin card sorting test (WCST)	20 correct, 12 errors 16 perseveration errors	Below average
Game of dice test (with 12 moves)	+800 € at finish	Thoughtful strategy
*Tendencies for malingering*		
Rey 15-Item Test	All correct	Inconspicuous
Test of memory malingering (TOMM)	Fist trial: 48 of 50	Inconspicuous
Test battery for forensic neuropsychology (TBFN)	13 correct, 2 false	Inconspicuous
Amsterdam short term memory test	Two errors in the first 15 trials	Inconspicuous

See details and references to the test battery listed in the Additional file 1: Supplementary online document S1

The reference group consisted of 10 male adults between 47 and 62 years (54.6 ± 4.3 years.; not differing in age from PR: t = − .8, *p* = .445; [35, 36]. All participants were right handed according to a modified version of the Edinburgh Handedness Questionnaire [37], and did not report psychiatric or neurological illness or psychotropic drug treatment. All participants were native German speakers holding a university degree. The participants were familiarized with the assessment environment and their participation was solely motivated by their interest in scientific investigations.

After the fMRI-session, verbal intelligence was examined in all participants with the MWT-B (Mehrfach–Wahl–Wortschatz-Test [38]), for which PR reached 130, and members of the reference group reached 126.0 ± 13.6 (test on normal distribution with Shapiro-Wilk-test: W = .89, *p* = .18). Here, PR did not differ from the group (t = − .28, *p* = .79 [35, 36]).

The study protocol was designed and performed according to the Helsinki Declaration (1964) and was approved by the Ethics Committee of Bremen University. All participants were informed about the procedure, and gave written consent to participate in the experiment.

### Task and stimuli

The tasks were presented visually. The experimental set-up includes two task conditions, a baseline (BASE) and a task of interest (TASK). Both task conditions included questions in order to test semantic memory performance. BASE-condition tested semantic memory about common knowledge such as for example “How is the head of the Catholic church called?”, and the TASK-condition tested semantic memory about public historical facts from contemporary history, for example “Which city hosted the Olympic Summer Games in 1996?”.

**Fig. 4:**
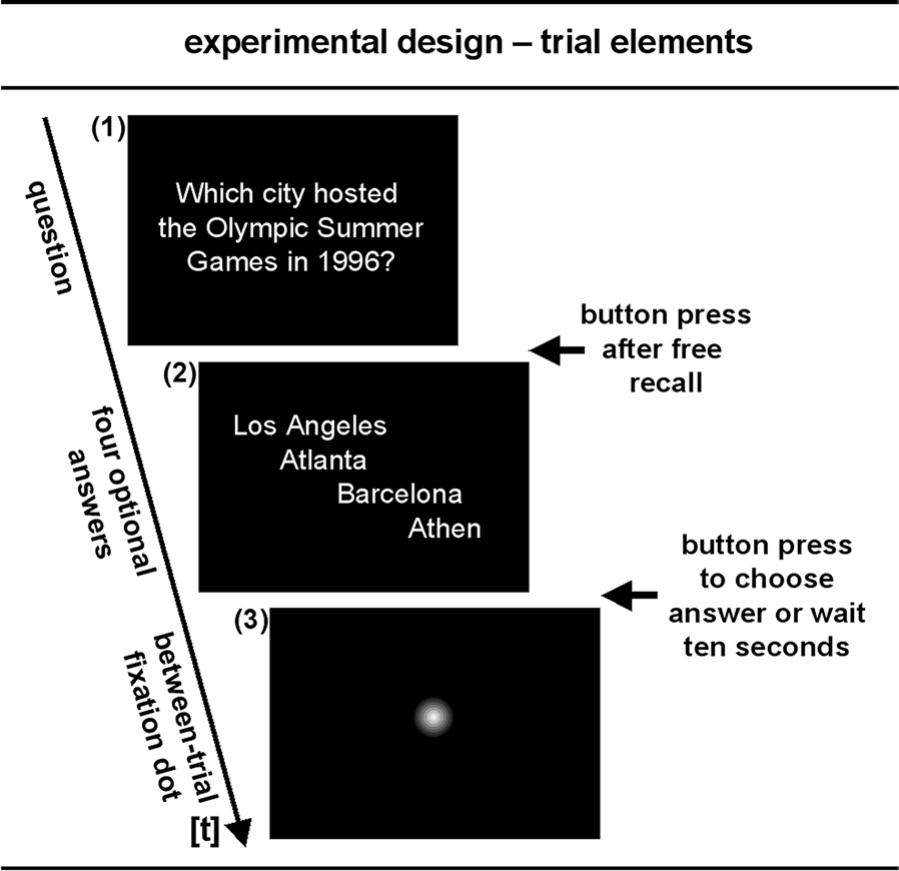
Experimental trial and trial elements: (*1*) Question, waiting for button press after free recall or conformation of omission, (*2*) choosing the correct answer after recall or recognition, or wait ten seconds for the between-trial period, and (*3*) between-trial fixation-period (3500 ± 500 ms, pseudo-randomised jittered)

The respective questions were presented via a digital projector on a mirror in the scanner tube in the center of the display as a centered text-block (see Fig. 4 for illustration). There was no time limit to think about the correct answer. Responses consisted of pressing the answer button with the right index finger. Individuals, however, were encouraged not to ruminate too long about the answer and they were asked to press a button with the right index finger, if they either believed to know the correct answer or when they were sure not to know the answer. This procedure was applied to ensure that participants produced free recall and not recognition performance. After pressing the button, four alternative answers (one correct solution) were presented slightly shifted one below the other in the center of the display (see Fig. 4 for illustration). If participants freely recalled the correct answer, they were asked to choose it from the four alternatives. If however, they did not recall the correct answer, but recognized it among the four alternatives, they were allowed to choose the correct one. If they neither recalled nor recognized the correct answer, participants were asked to wait (ten seconds) for the next trial. Between trials a fixation dot was presented for a pseudo-randomly jittered interval ranging between 3000 and 4000 ms.

After the MRI-scanner session, all questions were again presented by a paper–pencil-test. The participants were asked again to answer correctly to all BASE- and TASK-questions and to further provide detailed information about whether they freely recalled, recognized or did not know the correct answers during the fMRI-measurement. BASE- and TASK-trials were only included in behavioural and fMRI-data-analyses, if they were consistently answered correctly during both the fMRI-measurement and the paper–pencil-test, and furthermore, if they were labeled to be freely recalled and not just recognized. All other trials were neglected in behavioural data analyses and modeled as dummy-trials in the fMRI-analyses (see below).

50 BASE- and 48 TASK-trials were presented in a pseudo-randomized non-stationary probabilistic weighted sequence [39] during one experimental run of 17 ± 2 min.

### FMRI data acquisition and analyses

#### FMRI-data acquisition

Functional magnetic resonance imaging (fMRI) data were collected on a SIEMENS MAGNETOM scanner (Skyra syngo MR D13, 3 Tesla). The images were acquired using a BOLD weighted gradient echo echoplanar imaging (EPI) sequence (TE 30 ms). Forty four slices were acquired in interleaved order with no gap in axial orientation parallel to AC-PC with a GRAPPA accelleration factor of two leading to a TR of 2500 ms. The image volume covered the entire cerebrum and cerebellum. The in plane resolution was 3 × 3 mm^2^, the corresponding matrix  64 × 64, 411 ± 44 volumes were obtained during the functional run. Structural whole head T1 weighted images were acquired (TR/TE/TI/flip angle = 2400 ms/2.43 ms/900 ms/8°; matrix 256 × 256; slice thickness 1.0 mm; FOV 256 mm; 176 slices) for all participants.

#### FMRI-data analysis

Image analysis was performed using additional algorithms for the comparison of single individuals with group-related data (see below), which were implemented in SPM (http://www.fil.ion.ucl.ac.uk/spm/). For each session and participant, images were realigned to the first image in the time series to correct for head motion. The realigned images were spatially normalized into a standard stereotaxic space (Montreal Neurological Institute template) using a 12 parameter affine model. Dimensions after normalising procedures were 79(x) × 95(y) × 69(z) and resulting voxel size was 2 mm^3^. These spatially normalized images were smoothed to minimize noise and residual differences in gyral anatomy with a Gaussian filter set at 8.0 mm. Prior to the statistical analysis, a temporal high pass filter (250 s) was applied and global effects were removed. Pre-processed data sets were analysed using second-level random effects models [40] on the individual parameter estimates.

FMRI data were modelled for different trial element phases (see Fig. 4 for illustration): (1) from the start of task presentation until the first button press (separately for correct, incorrect, and omitted trials), (2) from the display of the four response alternatives to the second button press or until the trial time runs out (separately for correct, incorrect, and omitted trials), and (3) the fixation dot period between task trials resulting in 13 regressors in the design matrices. At the single-individual level, a t-contrast at each voxel for each participant was computed to produce statistical images for the contrast TASK- versus BASE-condition for the free recall trial element. At the second level, the resulting contrast images were used to identify the main task effect TASK versus BASE-condition by means of a one sample t-test (*p* < .001, uncorrected). For PR, TASK versus BASE-conditions were contrasted using t-statistics (*p* < .001, uncorrected). Percent signal change values for several regions of interest (ROIs) were extracted applying the software package Marsbar (Version 4.2 [41]). ROIs were extracted according to activation clusters resulting from the contrast “PR vs. reference group” related to “TASK vs. BASE”-condition contrasts. This was done to comprehensibly illustrate core aspects of the present data.

In order to inferentially compare brain activation patterns between PR and the reference group, we decided to follow the methods suggested by Crawford and colleagues [35, 42, 43]. The respective algorithms were implemented as an SPM compliant function that reads the specified individual contrast images and their respective design matrices (SPM.mat files). The appropriate beta images are then loaded as scores for the respective tasks and the calculus is performed. The resulting images were written to disk as spmT image files for use in the result function of SPM (*p* < .001, uncorrected) (see Fehr et al. [10] for further methodological details).

## Additional file

**Additional file 1.** Neural correlates of free recall of “famous events” in a “hypermnestic” individual as compared to an age- and education-matched reference group - supplementary information.

## Abbreviations

FMRI: functional magnetic resonance imaging
HS: hyperthymestic syndrome
LTM: long term memory
PR: protagonist
SPM: statistical parametric mapping
S–W-T: Shapiro–Wilk-test
