# Supplementary material for: Neural correlates of free recall of “famous events” in a “hypermnestic” individual as compared to an age- and education-matched reference group
Source: BMC Neurosci. 2018 Jun 19;19:35. doi: 10.1186/s12868-018-0435-y (PMC6006772; doi:10.1186/s12868-018-0435-y)
Supplement: Supplementary file 1 — Additional file 1. Neural correlates of free recall of “famous events” in a “hypermnestic” individual as compared to an age- and education-matched reference group - supplementary information. [file 12868_2018_435_MOESM1_ESM.doc]

Neural correlates of free recall of “famous events” in a “hypermnestic” individual as compared to an age- and education-matched reference group – supplementary information

Thorsten Fehr^1,2,3)^, Angelica Staniloiu^4,6)^, Hans J. Markowitsch^4,6)^, Peter Erhard^1,3,5)^, Manfred Herrmann^1,2,3)^

^1)^ Center for Cognitive Sciences, University of Bremen, Bremen, Germany

^2)^ Dpt. of Neuropsychology and Behavioral Neurobiology, University of Bremen, Bremen, Germany

^3)^ Center for Advanced Imaging, Universities of Bremen and Magdeburg, Germany

^4)^ Physiological Psychology, University of Bielefeld, Bielefeld, Germany

## ^5)^ AG in vivo MR, University of Bremen, Bremen, Germany

## ^6)^ Hanse Institute for Advanced Study (HWK), Delmenhorst, Germany

**- Supplementary Online Material –**

**Test battery for Protagonist PR:**

Protagonist PR was examined with a battery of different test inventories: Trail Making Test (TMT) [1,2], d2-R test [3], Wechsler-Memory-Scale-Revised (WMS-R) [4], Mehrfach-Wahl-Wortschatz-Test B (MWT-B) [5], Wechsler Intelligence Test [6], Rey-Osterrieth Figure Test (ROF) [7,1], Color-Word-Interference-Test (CWIT) [8,9], Verbal Learning Memory Test (VLMT) [10], Doors Test [11], Famous Faces Test [12], Extended Autobiographical Memory Test (EAMT) [13], Semantic old famous events (1970ies-1990ies) [12], Mind in the Eyes Test [14,15], Florida Affect Battery [16,17], Cronin-Golomb Concept Formation Task [18,19], Category Test [20], Tower of Hanoi [1,21,22], verbal fluency via Controlled Oral Word Association Test (COWAT; subscale of the Halstead-Reitan Neuropsychological Test Battery Test [23,24], Wisconsin Card Sorting Test (WCST) [25], Game of Dice Test [26,27], Rey 15-Item Test [1], Test of Memory Malingering (TOMM) [28,29,30], Test Battery for Forensic Neuropsychology (TBFN) [31], Amsterdam Short Term Memory Test [32,33].

**Additional references (test battery)**

1. Lezak MD. Neuropsychological assessment. Oxford: Oxford University Press; 1995.

2. Reitan R. Validity of the Trail Making Test as an indication of organic brain damage. Percept Motor Skill. 1958;8:271-6.

3. Brickenkamp R, Zillmer E. The d2 Test of Attention. Seattle, WA: Hogrefe and Huber; 1998.

4. Härting C, Markowitsch HJ, Neufeld H, Calabrese P, Deisinger K, Kessler J. Die Wechsler-Memory-Scale Revised [Wechsler Memory Scale – Revised]. Bern: Huber; 2000.

5. Lehrl S. Mehrfachwahl-Wortschatz-Intelligenztest (MWT-B) [Multiple Choice Lexis Intelligence Test]. Balingen: Spitta Verlag; 2005.

6. Dahl G. WIP-Reduzierter Wechsler-Intelligenztest [Abbreviated Wechsler Intelligence Test]. Meisenheim am Glan: Hain; 1972.

7. Osterrieth PA. Le test de copie d´une figure complex: Contribution à létude de la perception et de la mémoire [Test of copying a complex figure: Contribution to the study of perception and memory]. Arch Psychologie. 1944;30;286-356.

8. Bäumler G. Farbe-Wort-Interferenztest (FWIT) nach J. R. Stroop [Color-Word-Interference Test after J. R. Stroop]. Göttingen: Hogrefe; 1985.

9. Stroop JR. Studies of interference in serial verbal reactions. J Exp Psychol. 1935;18:643 - 62.

10. Helmstaedter C, Lendt M, Lux S. Verbaler Lern- und Merkfähigkeitstest (VLMT) [Verbal Learning and Memory Test]. Göttingen: Hogrefe; 2001.

11. Baddeley A, Emslie H, Nimmo-Smith I. Doors and People Test. Bury St. Edmunds, England: Thames Valley Test Company; 1994.

12. Fujiwara E, Brand M, Kracht L, Kessler J, Diebel A, Netz J, Markowitsch HJ. Functional retrograde amnesia: a multiple case study. Cortex. 2008;44:29-45.

13. Seidl U, Markowitsch HJ, Schröder J. Die verlorene Erinnerung. Störungen des autobiographischen Gedächtnisses bei leichter kognitiver Beeinträchtigung und Alzheimer-Demenz. In: Welzer H, Markowitsch HJ, editors. Warum Menschen sich erinnern können. [Why human beings can remember]. Stuttgart: Klett; 2006. p. 286-302.

14. Baron-Cohen S, Wheelwright S, Hill J, Raste Y, Plumb I. The "Reading the Mind in the Eyes" Test revised version: a study with normal adults, and adults with Asperger syndrome or high-functioning autism. J Child Psychol Psyc. 2001;42:241-51.

15. Reinhold N, Markowitsch HJ. Retrograde episodic memory and emotion: a perspective from patients with dissociative amnesia. Neuropsychologia. 2009;47:2197-206.

16. Bowers D, Blonder LX, Heilman KM. The Florida Affect Battery. Miami, FL: Florida University Press; 1991.

17. Breitenstein C, Daum I, Ackermann H, Lütgehetmann R, Müller E. Erfassung der Emotionswahrnehmung bei zentralnervösen Läsionen und Erkrankungen: Psychometrische Gütekriterien der "Tübinger Affekt Batterie". [Assessment of emotion perception in patients with lesions and diseases of the central nervous system: Psychometric validity criteria of the “Tubingen Affect Battery”]. Neurology & Rehabilitation. 1996;2:93-101.

18. Cronin-Golomb A, Rho WA, Corkin S, Growdon JH. Abstract reasoning in age-related neurological disease. J Neural Transm. 1987a;24:79-83.

19. Cronin-Golomb A, Rho WA., Corkin S, Growdon JH. Relational abilities in Alzheimer's disease and Parkinson's disease. Clin Neuropsychol. 1987b;1: 298.

20. Delis DC, Squire LR, Bihrle A, Massman P. Componential analysis of problem-solving ability: performance of patients with frontal lobe damage and amnesic patients on a new card sorting test. Neuropsychologia. 1992;30:683-97.

21. Borys SV, Spitz HH, Doans BA. Tower of Hanoi performance of retarded young adults and nonretarded children as a function of solution length and goal state. J Exp Child Psychol. 1982;33:87-110.

22. Spitz HH, Webster NA, Borys SV. Further studies of the Tower of Hanoi problem-solving performance of retarded young adults and non-retarded children. Dev Psychol. 1982;18:922-30.

23. Reitan RM, Wolfson D. The Halstead-Reitan Neuropsychological Test Battery. Tucson, AZ: Neuropsychology Press; 1995.

24. Golden CJ, Espe-Pfeifer P, Wachsler-Felder J. Neuropsychological Interpretation of Objective Psychological Tests. NY: Kluver; 2006.

25. Nelson HE. A modified card sorting test sensitive to frontal lobe defects. Cortex. 1976;12:313-24.

26. Brand M, Markowitsch HJ. Mechanisms contributing to decision-making difficulties in late adulthood: theoretical approaches, speculations and empirical evidence. Gerontology. 2010;56:435-40.

27. Brand M, Fujiwara E, Borsutzky S, Kalbe E, Kessler J, Markowitsch HJ. Decision-making deficits of Korsakoff patients in a new gambling task with explicit rules: associations with executive functions. Neuropsychology. 2005;19:267-77.

28. Tombaugh TN. Test of Memory Malingering (TOMM). New York: Multi Health Systems; 1996.

29. Teichner G, Wagner MT. The test of memory malingering (TOMM): normative data from cognitively intact, cognitively impaired, and elderly patients with dementia. Arch Clin Neuropsych. 2004;19:455-64.

30. Greiffenstein MF, Greve KW, Bianchini KJ, Baker WJ. Test of Memory Malingering and Word Memory Test: A new comparison of failure concordance rates. Arch Clin Neuropsych. (2008;23:801-7.

31. Heubrock D, Petermann F. Testbatterie zur Forensischen Neuropsychologie (TBFN) [Test battery for forensic neuropsychology (TBFN)]. Frankfurt: Swets and Zeitlinger; 2000.

32. Schagen S, Schmand B, de Sterke S, Lindeboom J. Amsterdam Short-Term Memory Test: a new procedure for the detection of feigned memory deficits. J Clin Exp Neuropsyc. 1997;19:43-51.

33. Schmand B, Lindeboom J. Amsterdam Short-Term Memory Test. Leiden, Netherlands. https://www.pits-online.nl/AKGT.html; 2012.
